# Hippocampus Radiomic Biomarkers for the Diagnosis of Amnestic Mild Cognitive Impairment: A Machine Learning Method

**DOI:** 10.3389/fnagi.2019.00323

**Published:** 2019-11-21

**Authors:** Qi Feng, Qiaowei Song, Mei Wang, PeiPei Pang, Zhengluan Liao, Hongyang Jiang, Dinggang Shen, Zhongxiang Ding

**Affiliations:** ^1^Department of Radiology, Affiliated Hangzhou First People’s Hospital, Zhejiang University School of Medicine, Hangzhou, China; ^2^Department of Radiology, Zhejiang Provincial People’s Hospital/People’s Hospital of Hangzhou Medical College, Hangzhou, China; ^3^GE Healthcare Life Sciences, Hangzhou, China; ^4^Department of Psychiatry, Zhejiang Provincial People’s Hospital/People’s Hospital of Hangzhou Medical College, Hangzhou, China; ^5^Department of Radiology and BRIC, University of North Carolina at Chapel Hill, Chapel Hill, NC, United States; ^6^Department of Brain and Cognitive Engineering, Korea University, Seoul, South Korea

**Keywords:** Alzheimer’s disease, amnestic mild cognitive impairment, magnetic resonance imaging, hippocampus, radiomics, machine learning

## Abstract

**Background**: Recent evidence suggests the presence of hippocampal neuroanatomical abnormalities in subjects of amnestic mild cognitive impairment (aMCI). Our study aimed to identify the radiomic biomarkers of the hippocampus for building the classification models in aMCI diagnosis.

**Methods**: For this target, we recruited 42 subjects with aMCI and 44 normal controls (NC). The right and left hippocampi were segmented for each subject using an efficient learning-based method. Then, the radiomic analysis was applied to calculate and select the radiomic features. Finally, two logistic regression models were built based on the selected features obtained from the right and left hippocampi.

**Results**: There were 385 features derived after calculation, and four features remained after feature selection from each group of data. The area under the receiver operating characteristic (ROC) curve, specificity, sensitivity, positive predictive value, negative predictive value, precision, recall, and *F*-score of the classification evaluation index of the right hippocampus logistic regression model were 0.76, 0.71, 0.69, 0.69, 0.71, 0.69, 0.69, and 0.69, and those of the left hippocampus model were 0.79, 0.71, 0.54, 0.64, 0.63, 0.64, 0.54, and 0.58, respectively.

**Conclusion**: Results demonstrate the potential hippocampal radiomic biomarkers are valid for the aMCI diagnosis. The MRI-based radiomic analysis, with further improvement and validation, can be used to identify patients with aMCI and guide the individual treatment.

## Introduction

Alzheimer’s disease (AD), the leading cause of senile dementia, is a degenerative disease of the central nervous system. Amnestic mild cognitive impairment (aMCI) refers to elderly individuals who do not meet the dementia standard but has episodic memory loss, which is considered the prodromal stage of AD (Morris et al., 2001). Recent studies have shown that AD prognoses are optimized when intervention occurs in the MCI phase, as opposed to after an AD diagnosis has been made (Jack et al., 2005; Plant et al., 2010; Prasad et al., 2011). Early and accurate diagnoses of aMCI are therefore of critical importance to the treatment of AD.

The hippocampus, an allocortical ridge of gray matter in the limbic system, plays a key role in cognition, especially in episodic memory (Spaniol et al., 2009; Lepage et al., 2015). As a highly central structure, it contains many gray matter nuclei and major white matter fiber bundles highly implicated in emotion, motor control, neuroendocrine activity, and memory. The hippocampus has been shown to be central to the AD pathological process (Braak and Braak, 1997), which is characterized by neurofibrillary tangles and amyloid-β plaques deposition. In parallel, recent studies establish the hippocampus as an invaluable region of interest (ROI) for diagnosis of AD. For example, structural brain MRI using voxel-based morphometry demonstrated hippocampal atrophy in AD and aMCI (Mcdonald et al., 2009; Fouquet et al., 2012). Some resting-state functional MRI studies have found abnormal functional connectivity between the hippocampus and other brain regions and in internal hippocampus in AD or MCI (Dennis and Thompson, 2014; de Flores et al., 2017; Sheng et al., 2017). In addition, diffusion tensor imaging studies indicated mean diffusivity abnormalities in the hippocampal region of patients with MCI (Fellgiebel et al., 2006; Mak et al., 2017).

Radiomics, a recently developed diagnosis and auxiliary detection technique, seeks to characterize pathologies using automated feature extraction algorithms to convert multi-modal ROI imaging data into a large number of features. These radiomic features encapsulate measures of shape, size, density, and texture of pathological tissues; population-level databases of radiomic features can be analyzed in order to improve diagnostic accuracy. As a data-driven general framework, the range of application of radiomics is broad. In the past, a radiomic analysis has been primarily applied to tumor diseases including glioma (Li et al., 2018), lung cancer (Tang et al., 2018), hepatocellular carcinoma (Cozzi et al., 2017), rectal cancer (Liu et al., 2017), oropharyngeal head and neck cancer (M. D. Anderson Cancer Center Head and Neck Quantitative Imaging Working Group, 2018), and breast cancer (Cameron et al., 2015). Recently, however, radiomics has also been applied to non-tumor diseases, such as autism spectrum disorder (Chaddad et al., 2017), attention deficit hyperactivity disorder (Sun et al., 2017), and xerostomia (Gabryś et al., 2018).

Artificial Intelligence Kit (A.K) is a platform geared toward accurate quantification and artificial intelligence. A.K software is a commercially available software developed by GE Healthcare, China. It has been registered and approved. It realizes several key steps of radiomics (data reading, image segmentation, feature calculation, feature selection, model building, and report generation) and has been employed in recent radiomics studies (Shao et al., 2018; Shu et al., 2019). A series of features are obtained by automating the analysis of target area heterogeneity for clinical diagnosis and prediction. In order to improve the early diagnosis of aMCI, a crucial step in AD intervention, we utilized A.K software to conduct a radiomic analysis of the hippocampus owing to its crucial role in AD and its well-established and unambiguous border.

## Materials and Methods

### Patient Population and Data Acquisition

Initially, 51 right-handed aMCI subjects were recruited from the Memory Clinic of Zhejiang Provincial People’s Hospital between September 2016 and March 2018, and 50 right-handed, volunteer normal control (NC) subjects were recruited from the health promotion center of the hospital. Informed consent was obtained from all subjects. The study protocol was approved by the local ethics committee of Zhejiang Provincial People’s Hospital (2012KY002). In addition, all methods were performed in accordance with the Declaration of Helsinki.

Laboratory tests, neuropsychological tests, physical examinations, and a brain MR scan were performed in subjects with aMCI. The criteria for identification of subjects with aMCI were as follows: (1) complaint of impaired memory; (2) maintaining normal performance; and (3) mini-mental state examination (MMSE) score >24 and ≤27 (Rivas-Vazquez et al., 2004).

The criteria for qualified NC subjects were as follows: (1) no neurological deficiencies, such as hearing or visual loss; (2) no neurological or psychiatric disorders, such as stroke, epilepsy, or depression; (3) no infarction, hemorrhage, or tumor lesion on conventional brain MR imaging; and (4) achieved an MMSE score ≥28.

The exclusion criteria were as follows: (1) stroke; (2) cerebral trauma; (3) other neurological disorders that cause memory impairment, such as brain tumor, Parkinson’s disease, and epilepsy; (4) systemic diseases, such as severe anemia, hypertension, and diabetes; (5) history of mental illness; and (6) high signal intensity lesions with a diameter of >5 mm using T2-FLAIR.

**Figure 1:**
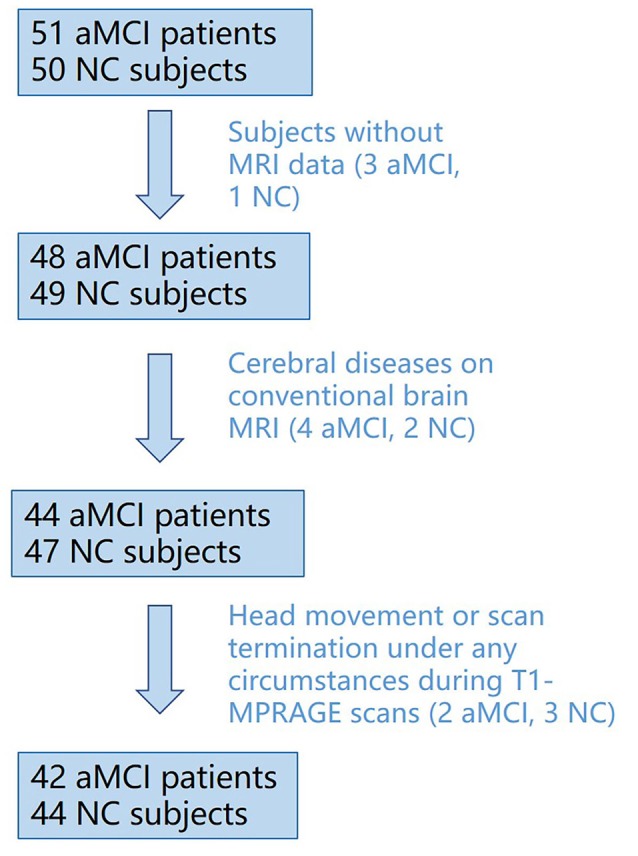
Flowchart of inclusion/exclusion process for subject recruitment.

From these recruited subjects, three aMCI patients and one NC subjects were excluded because of having no MRI data, and we excluded those who had obvious cerebral diseases on conventional brain MRI and had head movement or scan termination under any circumstances during T1-MPRAGE scans. Finally, 42 aMCI patients and 44 NC subjects were included, as shown in Figure 1.

All examinations were performed using an MR scanner (Discovery MR750 3.0T; GE Healthcare, Waukesha, WI, USA). In order to exclude the relative cerebral diseases according to the exclusion criteria, conventional brain MRI scans were performed first, including T1WI, T2WI, and T2-FLAIR. And then, three-dimensional (3D) T1-weighted magnetization-prepared rapid gradient echo (3D T1-MPRAGE) images were then collected. The scan parameters of the 3D T1-MPRAGE sequence are as follows: repetition time (TR) = 6.7 ms, echo time (TE) = 2.9 ms, inversion time (TI) = 450 ms, flip angle = 12°, field of view (FOV) = 256 × 256 mm^2^, slice thickness/gap = 1/0 mm, matrix = 256 × 256, and a total of 192 sagittal slices were collected. All collected data are from only one MR scanner.

**Figure 2:**
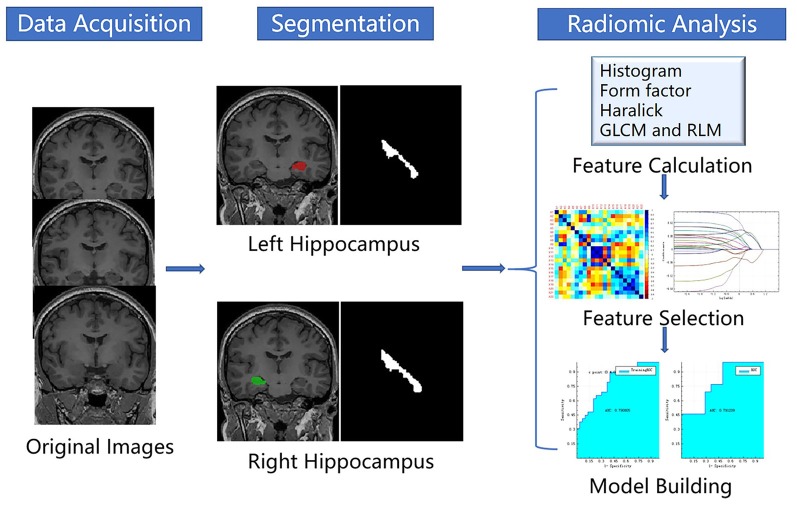
Flowchart of the radiomic process.

The process of a radiomic analysis was divided into five steps: (1) data loading; (2) segmentation; (3) feature calculation; (4) feature selection; and (5) machine learning. The radiomic process is shown in Figure 2.

### Segmentation

In this study, we use the structural T1-weighted MR images of the brain for a radiomic analysis based on A.K. We applied an efficient learning-based ROI method to segment the hippocampus (Wu et al., 2018). This method trains a joint classification and regression model to predict the location of the hippocampus; then, the prior hippocampal shape model is gradually deformed onto target image to adapt the target hippocampus.

### Feature Calculation

First, we imported the bulk of original 3D T1-MPRAGE data of the aMCI subjects and NC subjects into A.K software, and then we imported the bulk of the segmented left and right hippocampi. Then, we selected features for computation, including the histogram, form factor, gray level co-occurrence matrix (GLCM), Haralick, and gray level run-length matrix (RLM), in the “Parameters Choosing” window. Then, we chose options 1, 4, and 7 in the “Offsets of GLCM and RLM” window as the calculation based on the GLCM, and RLM was related to the offset. The histogram describing the distribution of image intensity in the hippocampus was analyzed. The form factor encapsulates the shape of the hippocampus. GLCM was characterized by statistical voxels of different directions and step of probability to get a co-occurrence matrix; and then to quantify the distribution of the co-occurrence matrix, the information such as the complexity of the lesion, the level of change, the thickness of texture was described (Dhruv et al., 2019). Haralick provides texture information of samples of GLCM from four directions (0°, 45°, 90°, and 135°) and an offset of 1 to calculate the sum of the means (Haralick et al., 1973). RLM is computed by counting the probability of successive occurrences of pixels with different directions and step length to obtain the matrix length to describe the complexity, the level of change, and the texture thickness of the lesion (Galloway, 1975; Chu et al., 1990). In total, 385 features were extracted for both the NC and aMCI groups.

### Feature Selection

First, we replaced abnormal values that cannot be calculated by mean. Next, we randomly set the proportion of training data and testing data to 0.7 and 0.3, respectively. Finally, we eliminated the unit limits for each column of feature by standardization. After preprocessing the extracted data, feature selection was done twice based on the calculated features of the left and right hippocampi. We performed the feature selection and modeling on the training data, and then we validated the model on the training data and testing data. Feature selection and dimension reduction are summarized by steps 1–3.

Step 1: we selected “*t*-test + rank test” as the method of dimensionality reduction method. The A.K software implemented the *t*-test to identify the features that contribute to the result (*P* < 0.05). The rank test was then used to select the features with significant difference from the preprocessed features of total data (*P* < 0.05). Then, the remaining features were integrated.

Step 2: we selected a correlation analysis to further reduce the dimension. We set the filter threshold to 0.9 and selected the Spearman rank correlation coefficient. The correlation analysis was done between any two feature columns. If the correlation coefficient between them exceeds 0.9, it indicates that the two features are highly correlated, and then one of them is randomly removed.

Step 3: finally, we adopted the least absolute shrinkage and selection operator (LASSO) regression model to identify the most useful features for the classification. This method added a penalty term on the objective function in the linear regression, that is, the L1 norm constraint of regression coefficients was set in a certain range, making some regression coefficients become zero, thus achieving the dimension reduction (Gui and Li, 2005). Here, cross-validation was used to obtain the best hyperparameters. We chose the λ, which meets the minimum criteria according to 10-fold cross-validation in the LASSO regression model. For the right hippocampus, the best λ value we chose was 1.0, whereas for the left, the best λ value was 0.6.

### Machine Learning

We shuffled the data and divided them into 0.7/0.3 train test. This procedure was repeated 10 times for the performance evaluation. We used machine learning method to build classification models for aMCI diagnosis. Specifically, we selected the logistic regression model and built two classification models on the basis of the selected features of the right and left hippocampi. This has set the result of linear function to an independent variable, which would be input into the sigmoid function. The classification for the probability *P* of the output was then determined by using a simple threshold of 0.5. The left and right hippocampus features and models were selected and built separately using A.K software. And the statistical methods used were the same. After first randomly setting the proportion of training data and testing data to 0.7 and 0.3 in preprocessing step, we then used the testing data to validate the trained logistic regression models.

## Results

### Comparison of Demographic and Neuropsychological Performance

The comparison of demographic variables between the aMCI patients and NC subjects showed no significant differences, which is performed by SPSS (version 22.0). Two-sample *t*-tests were used to assess group differences in age, education, and MMSE score between aMCI patients and NC subjects, whereas the chi-square test was used to assess group differences in gender. Neuropsychological performance was significantly different between these two groups (Table 1).

**Table 1:** Demographics performances of the aMCI and normal controls.

	aMCI group	NC group	Statistic	*p*-value
Sample size	42	44	NA	NA
Age (years, mean ± SD)	64.17 ± 10.57	65.43 ± 9.70	−0.58	0.56
Gender (male:female)	18:24	20:24	0.06*	0.81*
Education (years, mean ± SD)	7.74 ± 2.84	7.09 ± 3.38	0.96	0.34
MMSE	25.88 ± 0.92	29.14 ± 0.77	−17.92	0

Note. Unless otherwise indicated, statistics were calculated with *t*-tests. SD, standard deviation; MMSE, mini-mental state examination; aMCI, amnestic mild cognitive impairment; NC, normal controls. *x^2^ test was used.

### Feature Selection Results

**Figure 3:**
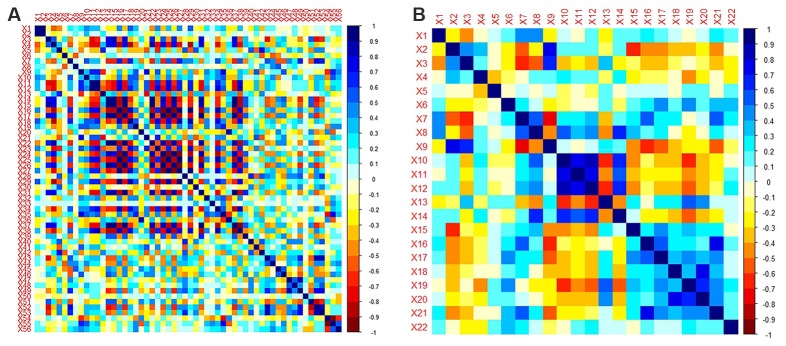
Correlation analysis graph of the right hippocampus **(A)** and left hippocampus **(B)**.

**Figure 4:**
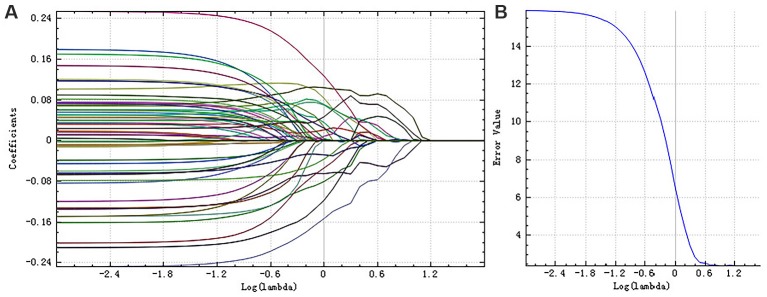
The coefficients-lambda graph **(A)** and the error-lambda graph **(B)** of the right hippocampus. We chose the λ corresponding to the lowest error rate.

**Figure 5:**
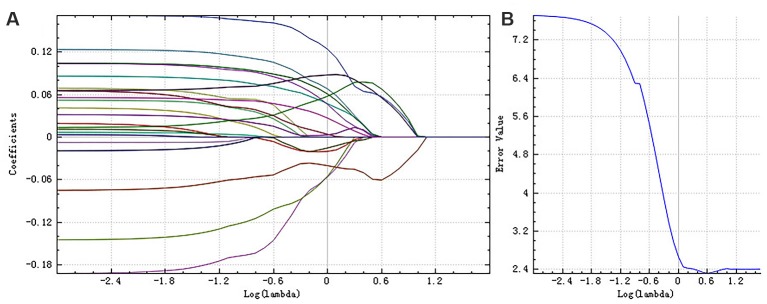
The coefficients-lambda graph **(A)** and the error-lambda graph **(B)** of the left hippocampus. We chose the λ corresponding to the lowest error rate.

A total of 385 features were extracted from the bilateral hippocampus. For right and left hippocampi, the remaining features following “*t*-test + rank test” were 104 and 35, respectively. After a correlation analysis, the remaining features were 56 and 22, respectively (see correlation analysis graph in Figure 3). Finally, the LASSO regression model yielded selection of four and four features for the right and left hippocampi, respectively (Table 2, Figures 4, 5).

**Table 2:** The remaining features after three steps of feature selection.

Remained features	The right hippocampus	The left hippocampus
Form factor	“Maximum 3D Diameter”	“Maximum 3D Diameter”
	“Surface Volume Ratio”
Histogram	“uniformity”	“uniformity”
RLM	“Long Run Emphasis_All Direction_offset7_SD”	“Low Gray Level Run Emphasis_All Direction_offset7_SD”
GLCM		“Inverse Difference Moment_All Direction_offset1_SD”

### Machine Learning Results

**Figure 6:**
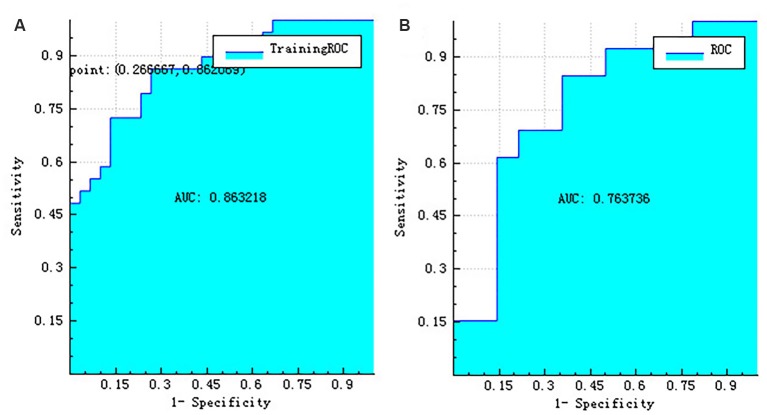
The ROC curve of training data **(A)** and the ROC curve of testing data **(B)** of the right hippocampus. ROC, receiver operating characteristic.

**Figure 7:**
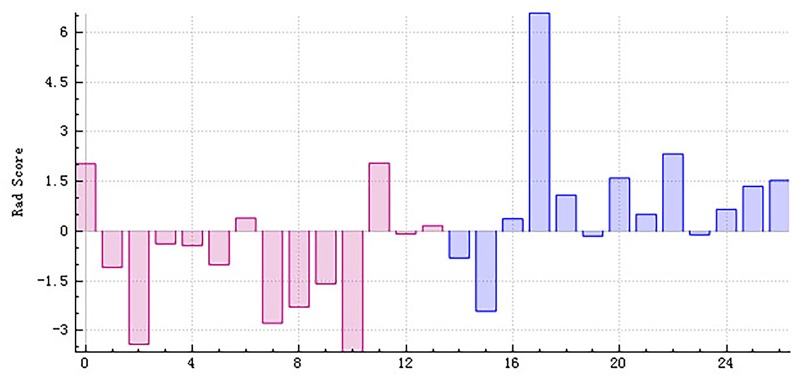
The radiomics score based on testing data of the right hippocampus. Red represents the normal group, and blue represents the patient group. The red area below the horizontal line and the blue area above the horizontal line represent the right prediction. On the contrary, the red area above the horizontal line and the blue area below the horizontal line represent the false prediction.

**Figure 8:**
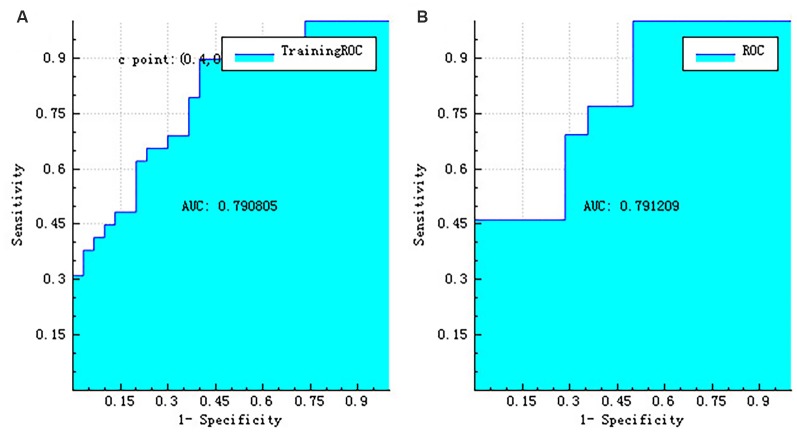
The ROC curve of training data **(A)** and the ROC curve of testing data **(B)** of the left hippocampus. ROC, receiver operating characteristic.

**Figure 9:**
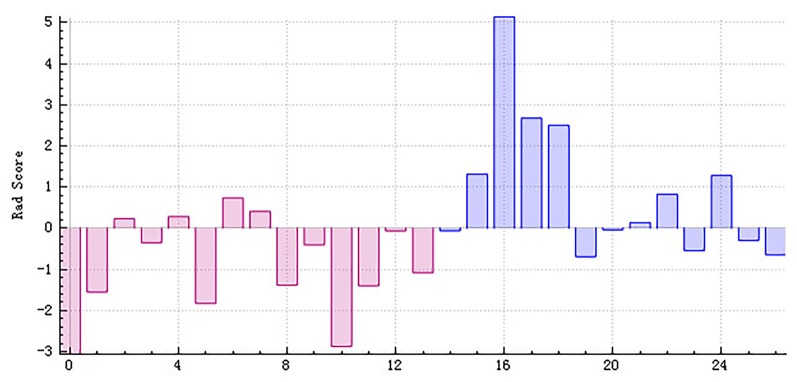
The radiomics score based on testing data of the left hippocampus. Red represents the normal group, and blue represents the patient group. The red area below the horizontal line and the blue area above the horizontal line represent the right prediction. On the contrary, the red area above the horizontal line and the blue area below the horizontal line represent the false prediction.

The right hippocampus and left hippocampus logistic regression models were performed to build classifiers that discriminate aMCI subjects from NC subjects using the four selected features for each hemisphere. For the right hippocampus model, the corresponding values of the area under the receiver operating characteristic (ROC) curve (AUC), specificity, sensitivity, precision, recall, and *F*-score were 0.76, 0.71, 0.69, 0.69, 0.69, and 0.69, respectively (Figures 6, 7). As the AUC of testing set is slightly lower than that of training set, overfitting exists in the model. And for the left hippocampus model, these predictive values were 0.79, 0.71, 0.54, 0.64, 0.54, and 0.58, respectively (Figures 8, 9). The close correspondence between the AUCs of the training set and the testing set indicates good fitting degree of the model.

## Discussion

With the goal of developing and improving early diagnosis of aMCI, closely related radiomic features of the hippocampus were identified and used to construct a logistic regression radiomic model of aMCI pathology. The two ROC curves indicate that the radiomic models had demonstrated moderate diagnostic value.

Feature selection is of critical importance to a radiomic analysis and thus represents an active area of development in the field. Typically, feature selection is achieved through statistical method, principal component analysis (PCA), and sequential forward selection. We use three statistical methods (*t*-test + rank test, correlation analysis, and LASSO regression model) in the study, all of which have been successfully employed in previous radiomic studies (de Oliveira et al., 2011; Moradi et al., 2015).

Classification and prediction are the ultimate goal of radiomics; a growing body of work demonstrates the efficacy of a radiomic analysis of non-tumor diseases in patient-disease prediction. Further, a texture analysis study found the texture differences in the corpus callosum and thalamus in patients with AD and aMCI (de Oliveira et al., 2011). Another texture analysis considered 3D texture as a possible diagnostic biomarker of AD (Zhang et al., 2012). In parallel, several studies have integrated machine learning methods into a radiomic analysis of AD: Zhang et al. (2012) believed that a 3D texture analysis could distinguish AD patients from the NC group, and the classification accuracy was between 64.3% and 96.4%. Beheshti et al. (2017) achieved MCI/NC classification accuracy of 70.38% using a support-vector machine (SVM) model, and Luk et al. (2018) predicted MCI conversion with 76.2% accuracy using a logistic regression model. The present work reinforces and expands upon these results, with our logistic models achieving AUC of 0.76 and 0.79 in distinguishing MCI from NC. Our work is potentially useful for clinical medicine because of its complete automation. In addition, we can obtain more information about the changes in microstructure after a radiomic analysis in aMCI patients.

There were four radiomics features selected in both the right and left hippocampi. The four major categories of features selected in this study respectively reflect the differences between aMCI group and NC group in hippocampal size and shape, gray value distribution, texture features, and spatial heterogeneity. The ratio of maximum 3D diameter and surface volume, the form factor parameters, was obtained, which described the 3D size and shape of the target regions; the larger the value of these two features, the larger the size of the hippocampus. Uniformity is one of the histogram parameters that are concerned with the properties of individual pixels and describes the distribution of voxel intensities within the image; this value is larger when the image is more complex. Inverse difference moment is one of the GLCM parameters. It describes the difference of lesion site, but the smaller the difference, the greater the value. Low gray level run emphasis and long run emphasis are considered as RLM parameters. They reflect the measurement of the nonuniformity of length and grayscale. The larger the value of low gray level run emphasis, the darker the lesion (i.e., the smaller the image gray value). And the larger the value of long-run emphasis, the smoother the image.

A recent comprehensive quantitative proteomic study on human hippocampus found that there were 372 proteins altering during the various stages of AD (Hondius et al., 2016). A structural MRI study indicated that the texture of hippocampus might serve as a neuroimaging biomarker for detecting early cognitive impairment (Sørensen et al., 2015). Some MRI studies demonstrated that the altered functional connectivity and the altered volume differences exist between the right and left hippocampi of MCI and AD (Peter et al., 2017; Sheng et al., 2017). Some studies reveal that the left hippocampus has greater atrophy than the right one in AD patients (Li et al., 2016). Furthermore, a study indicated that the left hippocampus atrophy had a stronger influence than the right in the prediction of aMCI (Zhang et al., 2011). In light of these results, we hypothesized that there might be some relationship between the altered protein expression and the radiomic features of the hippocampus in patients with aMCI, and thus, we built two independent hippocampus models for aMCI diagnosis.

There were several limitations to our study. First, although there was no statistically significant difference between groups in the age and sex ratio analysis, these two factors were not fully matched and may have slightly influenced the results. A complete 1:1 match in an age and sex ratio analysis may improve classification accuracy. Second, the diagnostic value of the radiomic model was not extremely high. Third, the sample size was limited, which could have influenced the performance of the radiomic models. Large-scale multicenter datasets are likely to improve the results of future radiomic studies.

The use of multi-modal imaging techniques, such as functional MRI and diffusion tensor MRI, improvements in genomics, and development of new models on the basis of both radiomic and clinical information such as MMSE and Montreal Cognitive Assessment (MoCA) scales also represent promising avenues for future research. A research breakthrough in the direction of imaging genomics is also needed.

## Conclusion

Our aMCI classification method based on hippocampal radiomic features represents an important and timely complement to MRI-based biomarkers of aMCI and AD. By benefit of its full automation, the method is particularly advantageous for clinical application. The MRI-based radiomic analysis, with further improvement and validation, can be used to identify patients with aMCI and guide the individual treatment.

## Data Availability Statement

The datasets generated for this study are available on request to the corresponding author.

## Ethics Statement

The studies involving human participants were reviewed and approved by Ethics Committee of Zhejiang Provincial People’s Hospital (2012KY002). The patients/participants provided their written informed consent to participate in this study.

## Author Contributions

ZD, DS, and QF designed the experiments. QF, QS, MW, ZL, and HJ performed the experiments and analyzed the data. ZD, QF, and PP interpreted the results and drafted the manuscript. All the authors read and approved the final version of the manuscript.

## Conflict of Interest

PP was employed by GE Healthcare Life Sciences.

The remaining authors declare that the research was conducted in the absence of any commercial or financial relationships that could be construed as a potential conflict of interest.
